# *χ*2-BidLSTM: A Feature Driven Intrusion Detection System Based on *χ*2 Statistical Model and Bidirectional LSTM

**DOI:** 10.3390/s22052018

**Published:** 2022-03-04

**Authors:** Yakubu Imrana, Yanping Xiang, Liaqat Ali, Zaharawu Abdul-Rauf, Yu-Chen Hu, Seifedine Kadry, Sangsoon Lim

**Affiliations:** 1 School of Computer Science and Engineering, University of Electronic Science and Technology of China (UESTC), Chengdu 611731, China; yakubu.imrana@uds.edu.gh (Y.I.); xiangyanping@gmail.com (Y.X.); 2 Department of Electrical Engineering, University of Science and Technology, Bannu 28100, Pakistan; engr.liaqat@ustb.edu.pk; 3 Department of Education, University for Development Studies (UDS), Tamale P.O. Box TL 1350, Ghana; iris.zahra01@gmail.com; 4 Department of Computer Science Information Management, Providence University, Taichung City 433, Taiwan; ychu@pu.edu.tw; 5 Department of Applied Data Science, Noroff University College, 4612 Kristiansand, Norway; seifedine.kadry@noroff.no; 6 Department of Computer Engineering, Sungkyul University, Anyang 14097, Korea

**Keywords:** deep learning, feature selection, intrusion detection systems, chi-square, bidirectional LSTM

## Abstract

In a network architecture, an intrusion detection system (IDS) is one of the most commonly used approaches to secure the integrity and availability of critical assets in protected systems. Many existing network intrusion detection systems (NIDS) utilize stand-alone classifier models to classify network traffic as an attack or as normal. Due to the vast data volume, these stand-alone models struggle to reach higher intrusion detection rates with low false alarm rates( FAR). Additionally, irrelevant features in datasets can also increase the running time required to develop a model. However, data can be reduced effectively to an optimal feature set without information loss by employing a dimensionality reduction method, which a classification model then uses for accurate predictions of the various network intrusions. In this study, we propose a novel feature-driven intrusion detection system, namely χ2-BidLSTM, that integrates a χ2 statistical model and bidirectional long short-term memory (BidLSTM). The NSL-KDD dataset is used to train and evaluate the proposed approach. In the first phase, the χ2-BidLSTM system uses a χ2 model to rank all the features, then searches an optimal subset using a forward best search algorithm. In next phase, the optimal set is fed to the BidLSTM model for classification purposes. The experimental results indicate that our proposed χ2-BidLSTM approach achieves a detection accuracy of 95.62% and an F-score of 95.65%, with a low FAR of 2.11% on NSL-KDDTest^+^. Furthermore, our model obtains an accuracy of 89.55%, an F-score of 89.77%, and an FAR of 2.71% on NSL-KDDTest^−21^, indicating the superiority of the proposed approach over the standard LSTM method and other existing feature-selection-based NIDS methods.

## 1. Introduction

In present-day society, various organizations and individuals have become more and more reliant upon information and communication technology (ICT), due to the increasing number of useful technologies. The rise in reliance has resulted in a greater demand for more stable and reliable ICT components and services. As a section of ICT, the Internet provides a medium for individuals and organizations to accomplish tasks in their everyday lives. However, as the data flow and the information traffic over the Internet increase, user privacy and transactions become more prone to malicious users’ threats and attacks (intrusions). An intrusion is a succession of activities aiming to jeopardize the security of a network system [1].

Intrusion detection systems (IDSs) have proven essential in the security domain and play a vital role in detecting different types of malicious behaviors and attacks. IDSs can be grouped into three basic strategic concepts (misuse detection, anomaly detection, and a hybrid of the two) [2,3]. Misuse detection is a signature-based approach used to identify a particular matching behavior or signature, compare it to recorded user behavior or activities, and raise a signal [4,5,6]. Anomaly detection is used to spot activities that are significantly different from normal user activity. In anomaly detection systems, an action is raised if there is some deviation from a predefined computer state [7,8,9]. Hybrid detection is a fusion of anomaly and misuse detection methods used to identify malicious activities [2,10,11]. It is vital to mention that network intrusion or attacks can come from outside the network (outsider attacks) or from within the network (insider attacks). Researchers have proposed several different intrusion detection systems over the past few decades using machine learning, deep learning, and other statistical methods. However, in recent times, machine learning and deep learning techniques have gained more attention in many different research areas, including intrusion detection [12]. They have become the most commonly adopted approaches for many intrusion detection systems (IDS).

In the literature, machine learning methods such as support vector machine (SVM), decision trees (DT), k-nearest neighbor (KNN), artificial neural networks, and deep neural networks (DNN) have been widely used for the detection of network intruders [13,14,15,16]. However, the performance of these techniques depends heavily on simulated datasets. These datasets often require many features for training, making them computationally expensive for most classification models. Furthermore, using large numbers of features may result in low performance, because some features may be redundant and irrelevant to the performance of a model.

Therefore, it is necessary to perform feature selection before training, to eliminate redundant and irrelevant features from the datasets. Feature selection plays an important role in data preprocessing for most machine learning models. It is the process of selecting features with the highest contributions to the predictive variable. Feature selection can be performed manually or using algorithms (automatically) to reduce the dimensions of the data to a subset of features relevant to building a predictive model. There are three main categories of feature selection in the literature: wrapper, filter, and hybrid methods [17]. The wrapper method utilizes the greedy search strategy to evaluate all possible feature combinations against a criterion for evaluation based on machine learning algorithms [17,18]. The filter method, on the other hand, is not dependent on any machine learning algorithm. Features are selected based on the variable characteristics or intrinsic properties, which are measured via statistical analysis [19,20]. A hybrid or embedded method uses a combination of the properties of wrapper and filter methods [17,21]. Motivated by the positive impact of feature selection on the performance of machine learning and deep learning models for several different problems, we have developed a new IDS called $\chi^{2}$-BidLSTM for network systems.

### Main Contributions

The proposed $\chi^{2}$-BidLSTM IDS integrates $\chi^{2}$ with a BidLSTM-based deep learning model. The $\chi^{2}$ statistical model is used for the ranking and selection of features based on their $\chi^{2}$ test scores. The selected optimal features are used to train a bidirectional long short-term memory (BidLSTM)-based recurrent neural network (RNN) for network intrusion detection. The NSL-KDD dataset, which can be accessed via the University of New Brunswick (UNB) data repository, is used to train and evaluate our $\chi^{2}$-BidLSTM model’s performance. The contributions of this paper are as follows:

1. Developing and implementing an intrusion detection system based on a bidirectional long short-term memory integrated with a $\chi^{2}$ feature selection model.
2. To the best of our knowledge, no prior work has addressed the hybridization of the bidirectional LSTM model with $\chi^{2}$ statistical model for network intrusion detection.
3. The $\chi^{2}$-BidLSTM method uses fewer features for training and testing purposes, and thus reduces the complexity of traditional BidLSTM and also improves its classification accuracy.
4. A better classification accuracy than the traditional bidirectional LSTM model is obtained. Additionally, our approach outperforms existing state-of-the-art methods in the literature.

The remainder of this work is organized as follows. Section 2 presents a review of related work in the literature. A description of the dataset and the proposed methodology is presented in Section 3. Section 4 presents the implementation, experimental results, and discussion. Section 5 discusses the model complexity and limitations. In Section 6, we present the conclusions and future directions of the study.

## 2. Related Work

As an essential element for ensuring security in network systems, IDSs continuously draw the interest of many researchers. Many models have been developed to enhance the effectiveness of IDSs in network systems. In this section, we discuss the literature related to IDS techniques based on machine learning (ML) and deep learning (DL) that leverage feature selection for network anomaly detection.

The authors in [22] proposed a hybrid IDS approach using the NSL-KDD dataset, which focuses on combining the probability distributions of different learning algorithms using information gain (IG) and a voting algorithm to select relevant features for classification. The hybrid method comprises the J48, Random Tree, Meta Pagging, REPTree, Decision Stump, AdaBoostM1, and naive Bayes base classifiers. Although the technique demonstrated a good performance of 99.81% and 98.56% accuracy for binary and multi-class problems, respectively, there are still some concerns that need attention. The feature selection process in this approach is often biased towards variables with distinct values, not variables that have observations with large values, which can result in over-fitting and poor performance. In [23], Hota and Shrivas also developed a framework that utilizes different feature selection methods for irrelevant feature removal. The findings suggested that the C4.5 algorithm could obtain the greatest accuracy with IG for just 17 features of the NSL-KDD dataset. The study investigated the performance of four different feature selection methods (i.e., correlation, information gain, relief, and symmetrical uncertainty) integrated with the C4.5 decision tree algorithm for classification. According to the experimental findings, the most efficient amongst the four selection methods was information gain with C4.5, which obtained a detection accuracy of 99.68%. Although the result is promising, the method tends to be skewed towards attributes with many possible values, leading to poor generalization. Moreover, the entropy model employed in C4.5 has many time-consuming logarithmic operations, sorting operations, and continuous values resulting in high computational cost. Using logistic regression combined with a search strategy, the authors in [24] presented a feature-selection-based IDS model that selects the best subset of features from the KDDCUP’99 and the UNSW-NB15 datasets. The findings indicated that their algorithm yields a good detection accuracy with just 18 selected features from the KDDCUP’99 dataset and 20 selected features from the UNSW-NB15 dataset.

Acharya and Singh [25] proposed a novel bio-driven feature selection approach that utilizes the Intelligent Water Drops algorithm combined with an SVM classifier for network intrusion detection. Their approach, also known as a swarm optimization algorithm, produced a high performance on the KDDCUP’99 dataset. The results indicated that the approach obtained a high accuracy of 93.12%, a detection rate of 91.35%, and a reduced false alarm rate of 3.35%, compared to other methods. The authors in [26] introduced a hybrid IDS mechanism that integrates feature selection and clustering using SVM and K-medoids clustering strategies. In this approach, the authors trained a naïve Bayes classifier on the KDDCUP’99 dataset. They evaluated the model using the detection rate, accuracy, and false alarm rate. The evaluation results indicated that the proposed approach obtained a higher detection rate of 90.1%, an accuracy of 91.5%, and a false alarm rate of 6.36%. In [27], Jabbar et al. presented a cluster-oriented ensemble model for network intrusion detection. The model was developed using the alternating decision tree technique (ADTree) and the K-nearest neighbor (KNN) algorithm. In experiments, their proposed approach showed a better performance with regard to accuracy and detection rate, compared to other methods in the literature.

In another approach [28], Paulauskas and Auskalnis introduced an ensemble IDS model. The model was developed using naïve Bayes (NB), C5.0, J48, and the partial decision list algorithms as base classifiers, with the notion of integrating multiple learners. Experimental findings indicated that the approach obtained better accuracy for network intrusion detection. To combat the high-dimensionality problem in network traffic, Zhou and Cheng [29] developed a heuristic feature selection algorithm known as the correlation-based feature selection bat algorithm (CFS-BA). Their strategy obtains the best feature subset by evaluating the correlations among features. The authors further built an ensemble model that incorporates random forest, forest-oriented penalizing attribute, and C4.5 decisions, using the rule of the average of probabilities (AoP). The model was trained and evaluated using CIC-IDS2017, KDDCUP’99, and the NSL-KDD datasets. The results showed that the CFS-BA ensemble approach produced a better performance, compared to other existing methods.

In [30], Pham et al. presented a hybrid approach that leverages gain ratio and bagging techniques for network intrusion detection. The former (gain ratio) is utilized to obtain the best features. The latter (bagging) is used to integrate tree-based core classifiers. The approach was evaluated using the NSL-KDD dataset. The results showed that the bagging method combined with J48 as the core classifier produced better performance for 35 features. The authors in [31] proposed a wrapper-based IDS that utilizes a hyper-graph (HG) and a genetic algorithm (GA) for producing possible subsets of features. The approach uses SVM as a classification algorithm, which is evaluated on the NSL-KDD dataset. From the evaluation, their proposed method exhibited a performance accuracy of 96.72% with 35 selected features.

In [32], Abdullah et al. developed an IDS model based on splitting the data input into several subsets relative to the attack types. In this work, IG was used to select the best features for each subset. Using random forest (RF) and partial decision list (PART) as core classifiers, the method was evaluated on the NSL-KDD dataset. Experimental findings illustrated that higher accuracy was achieved with the RF and PART classifiers combined with product probability. In [33], Mohammadi et al. introduced a feature-selection-based IDS that incorporates a clustering algorithm. The methodology was developed using a wrapper method that leverages a linear correlation coefficient (LCC) algorithm and a filter strategy that utilizes a cuttlefish algorithm (CFA). Their approach trained a decision tree (DT) classifier on the KDDCUP’99 dataset. Experimental results with 10-fold cross-validation showed that the method obtained a 95.03% accuracy, a 95.23% detection rate, and a reduced false alarm rate of 1.6%. The authors in [34] developed a hybrid intrusion detection system that integrates principal component analysis (PCA) and information gain (IG) algorithms for feature selection. Their approach was evaluated on the NSL-KDD, Kyoto 2006+, and ISCX 2012 datasets using three ensemble classifiers (i.e., multi-layer perceptron (MLP), SVM, and instance-based learning algorithms (IBK)). The IG-PCA method exhibited more remarkable performance in detection rate, accuracy, and false alarm rate than other existing strategies.

Using the organic combination of several deep learning methods, the authors in [35] proposed a novel anomaly detection approach known as HELAD. The authors first performed feature extraction and selection using the damped incremental statistics algorithm (DISA). An autoencoder was then trained with selected features of a label dataset while noting the irregular (abnormal) score labels in the data traffic. They further trained an LSTM model using the irregular score label and obtained the final score using a weighted technique. In the experiment, the HELAD method produced a better accuracy compared to other state-of-the-art methods. In [36], the authors used a multi-objective technique to obtain the best subsets of features, which were then evaluated based on three decision tree algorithms (i.e., NB, RF, and C4.5). The three algorithms were trained and tested using the CIC-IDS2017, UNSW-NB15, and NSL-KDD datasets. In the experiment, the NSGA2-LR approach showed promising results compared to other methods.

## 3. Materials and Methods

In this section, we present a detailed description of the dataset used in our study and the proposed methodology.

### 3.1. Description of Dataset

One of the benchmark datasets utilized by researchers on intrusion detection in the security domain is the NSL-KDD dataset [37]. It is publicly available in the online data repository of the University of New Brunswick (UNB) [38]. The NSL-KDD dataset is a modified form of the KDDCUP’99 dataset presented in [39]. The proposed model was trained and evaluated on the NSL-KDD dataset. We selected this dataset because of the following advantages:

1. The dataset has a reasonable and sufficient number of traffic records that can be used to perform the study.
2. It does not contain redundant traffic in the training set, ensuring that classifiers are not biased toward more frequently occurring records.
3. The testing set has no duplicate records; hence, the performance of learning algorithms is not biased by models with higher detection rates on more frequently occurring records.
4. The fraction of records in the main KDD dataset is inversely proportional to the overall records chosen from each difficulty level category. Therefore, the prediction rates of various ML algorithms differ over a greater range, making accurate evaluation of various learning methods more effective.

The dataset includes a training set (i.e., KDDTrain^+^) containing 125,973 data records and two different test sets (i.e., KDDTest^−21^ and KDDTest^+^) containing 11,850 and 22,544 data records, respectively, as presented in Table 1.

### 3.2. Data Preprocessing

As presented in Table 2, the NSL-KDD dataset has forty-one (41) features, of which three are non-numeric. The non-numeric features are service, protocol_type, and flag. The dataset has one classification label that can be categorized into two classes (i.e., normal and attack) for a 2-class classification or five classes for a 5-class classification. The five classes include Remote-2-Local (R2L), User-to-Root (U2R), Denial of Service (DoS), Probe, and Normal. Apart from the normal class, the remaining four classes represent the different attack types found in the dataset (see Table 3). Like any neural network model, the proposed approach uses only numeric values as inputs. Hence, we converted all the non-numeric data inputs to numeric form by encoding and assigning unique integer values to each of them. As an integral part of data preprocessing, normalization plays an essential role in producing a balanced dataset. The commonly used normalization strategies in machine learning and data science include decimal scaling, z-score, and min-max. The NSL-KDD dataset exhibits uneven distribution for some features (e.g., *src_bytes* and *dst_bytes*), leading to biased results. To ensure that the proposed model does not produce biased results, we transformed all 41 features to values within the range of 0 to 1 by utilizing the min-max feature scaling technique, as shown in Equation (1):

(1) $$x=\frac{z-z_{min}}{z_{max}-z_{min}}$$

where *z* signifies the original value of the feature and *x* represents the newly scaled number.

### 3.3. Proposed Approach

**Figure 1:**
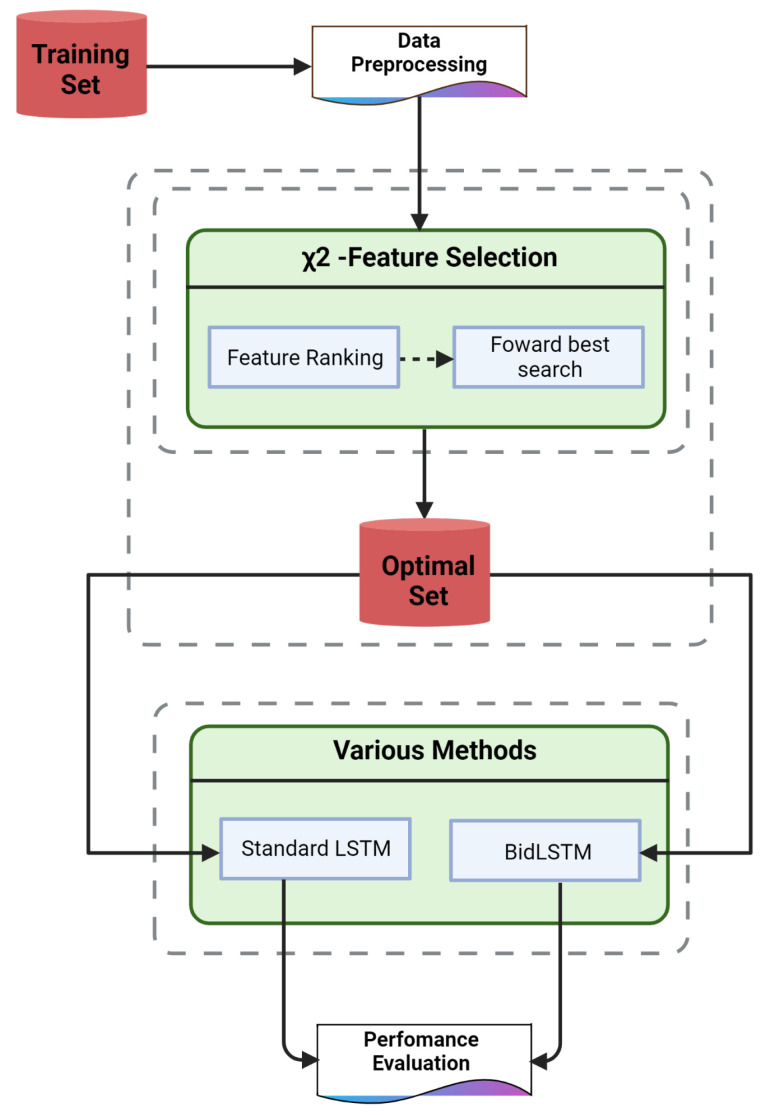
The proposed IDS architecture.

The proposed method (see Figure 1) in this paper is chi-square bidirectional long short-term memory ($\chi^{2}$-BidLSTM), which involves two steps. The first step utilizes a chi-square statistical model to select optimal features from the dataset. The second step trains a bidirectional LSTM predictive model on the optimal set.

#### 3.3.1. Chi-Square ($\chi^{2}$) Feature Selection

A $\chi^{2}$ model computes the $\chi^{2}$ statistics for every feature ($F_{i}$) and class (θ) to measure the level of independence between each class and feature. It also indicates features that are most likely to be irrelevant (not class-dependent) for classification [40]. The feature selection process first partitions the data and ranks the features, and then performs a search to obtain an optimal subset from the ranked set of features [41]. The features are ranked using the $\chi^{2}$ test scores. For instance, consider a 2-class (i.e., Normal and Attack) classification with *m* instances. We can construct a table to obtain the $\chi^{2}$ test scores, as shown in Table 4.

Here, φ represents the total number of instances with feature $F_{i}$, and the total number of instances without $F_{i}$ is represented by m−φ. In addition, δ denotes the total number of normal instances. The total number of attack instances is denoted by m−δ. The $\chi^{2}$ test statistic compares the observed values (*O*) measured from the data with the expected values (*E*). From Table 4, the observed values are *n*, *a*, ν, and ρ. Let $E_{n}$, $E_{a}$, $E_{\nu }$, and $E_{\rho }$ represent their expected values, respectively. Using the assumption that the two occurrences are independent, we can compute the expected values as:

(2) $$E_{n}=\left(n+a\right)\left(\frac{n+a}{m}\right)$$

Similarly to Equation (2), we can also compute the expected values $E_{a}$, $E_{\nu }$, and $E_{\rho }$. The $\chi^{2}$ test statistic for a goodness-of-fit test is generally obtained by:

(3) $$\chi^{2}=\sum_{i=1}^{k}\frac{{\left(O_{i}-E_{i}\right)}^{2}}{E_{i}}$$

where *i* is the number of different data classes, *O* denotes the observed values measured from the data, and *E* represents the expected values. We can therefore compute the $\chi^{2}$ test statistic for Table 4 as:

(4) $$\chi^{2}=\frac{{\left(n-E_{n}\right)}^{2}}{E_{n}}+\frac{{\left(a-E_{a}\right)}^{2}}{E_{a}}+\frac{{\left(\nu -E_{\nu }\right)}^{2}}{E_{\nu }}+\frac{{\left(\rho -E_{\rho }\right)}^{2}}{E_{\rho }}$$

The test statistic in Equation (4) is used to rank the features. Subsequently, we perform a forward best-first search to select the features with the highest test scores as the optimal set. Thus, we first select a feature with the highest $\chi^{2}$ test result and check its performance using the BidLSTM model. Another feature is added to the subset of features in the subsequent iteration based on the test score. Again, we investigate the performance of the subset of features with the BidLSTM model. This procedure is repeated until every ranked feature is added to the subset. The subset of features with the best performance is then selected as the optimal set and supplied to the BidLSTM predictive model to produce the best performance results.

#### 3.3.2. BidLSTM Model

In this subsection, we present the deep learning methods used in this study. We give a brief overview of the working principles of RNNs in general and narrow it down to the main methods: the LSTM and the BidLSTM.

**Figure 2:**
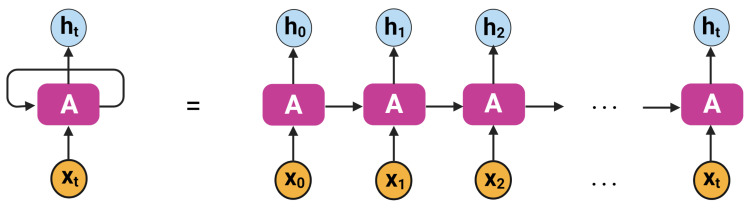
An unrolling RNN architecture [46].

RNN is a generalized form of traditional feed-forward network with internal memory, capable of propagating information from the past to the future. It generates loops in the networks which enable information to persist. The loops are utilized together with the memory state to process a sequence of input data [42]. RNN is a category of DNN that is able to utilize previous outputs while maintaining hidden layers that serve as storage for information [43,44]. The same weights and biases are supplied to all layers, thereby minimizing the challenge of memorizing and increasing parameters. The basic architecture of an RNN is shown in Figure 2. RNNs may be suitable for solving several research problems, but they suffer from the drawback of vanishing gradients, which inspired the development of LSTM in [45].

**Figure 3:**
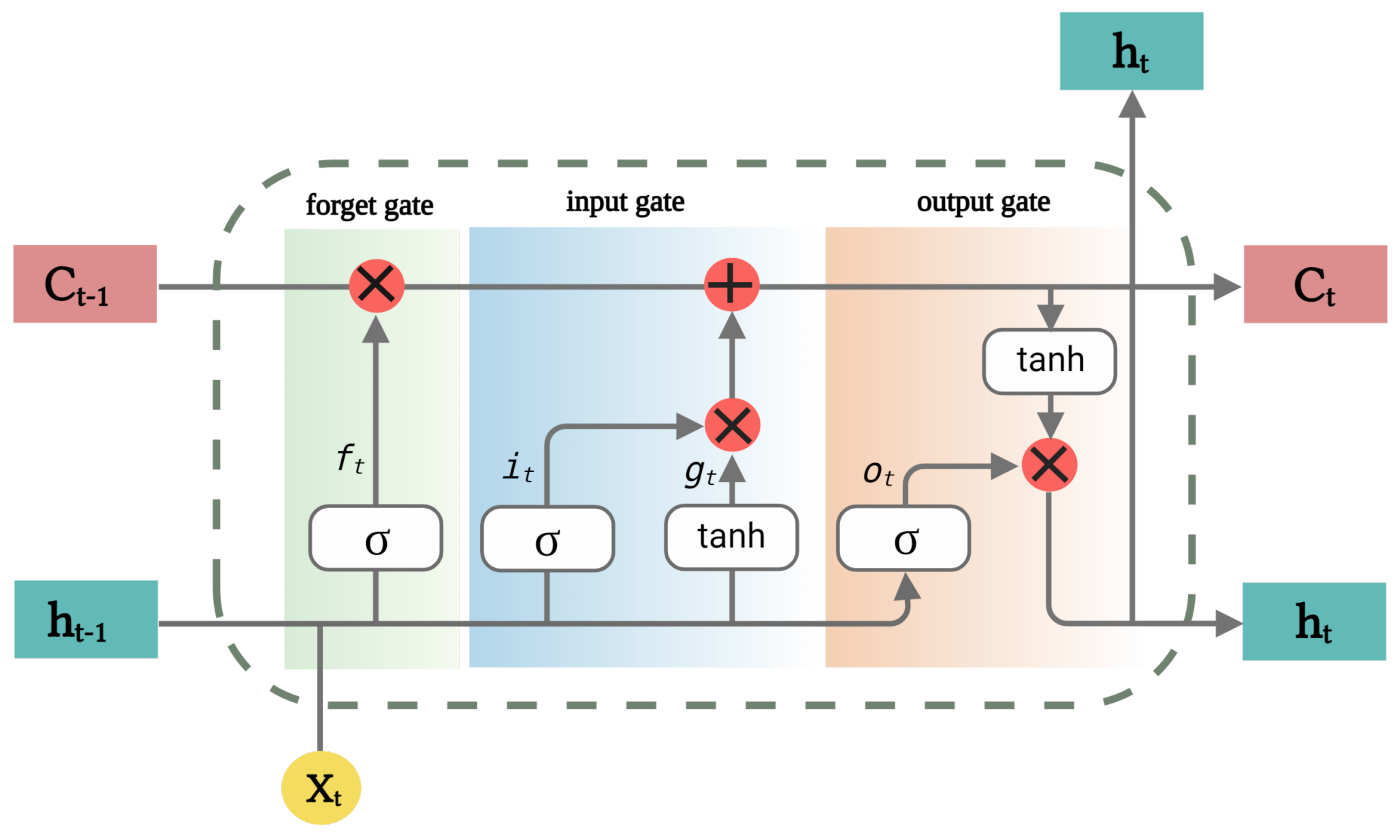
The LSTM memory cell.

The LSTM network is a more advanced version of the RNN that learns long-term dependencies via a gating mechanism. It is a solution to the vanishing gradients problem encountered when training conventional RNNs [47]. The gates and cell state are the LSTM network’s basic principles. The cell state is considered as the network’s memory and serves as route to propagate relevant information. The gates (i.e., forget, input, and output gates) control the information flow and determine what knowledge should be kept or discarded (forgotten), as shown in Figure 3. Equations (5)–(9) give the expressions for the cell state and various gates at the periods *t* and t−1, as follows:

(5) $$i_{T}=\sigma \left[\left({\hat{W}}_{\alpha i}*\alpha_{T}\right)+\left({\hat{W}}_{\beta i}*\beta_{T-1}\right)+\left({\hat{W}}_{\zeta i}*\zeta_{T-1}\right)+\lambda_{i}\right]$$

(6) $$f_{T}=\sigma \left[\left({\hat{W}}_{\alpha f}*\alpha_{T}\right)+\left({\hat{W}}_{\beta f}*\beta_{T-1}\right)+\left({\hat{W}}_{\zeta f}*\zeta_{T-1}\right)+\lambda_{f}\right]$$

(7) $$\zeta_{T}=\left(f_{T}*\zeta_{T-1}\right)+i_{T}\tanh\left[\left({\hat{W}}_{\alpha \zeta }*\alpha_{T}\right)+\left({\hat{W}}_{\beta \zeta }*\beta_{T-1}\right)+\lambda_{\zeta }\right]$$

(8) $$o_{T}=\sigma \left[\left({\hat{W}}_{\alpha o}*\alpha_{T}\right)+\left({\hat{W}}_{\beta o}*\beta_{T-1}\right)+\left({\hat{W}}_{\zeta o}*\zeta_{T-1}\right)+\lambda_{o}\right]$$

(9) $$\beta_{t}=o_{T}\tanh\left(\beta_{T}\right)$$

where $i_{T}$ denotes the input gate, α represents the input vector, $o_{T}$ is the output gate, $\beta_{T}$ denotes the output, and $f_{T}$ represents the forget function. The cell state is given by ζ, with $\hat{W}$ and λ as the weight and bias parameters, respectively.

**Figure 4:**
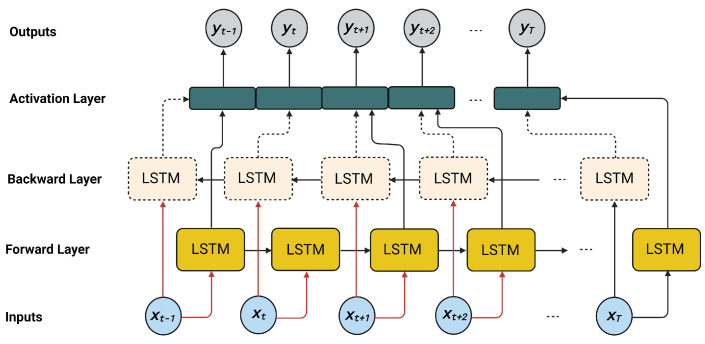
A bidirectional LSTM architecture.

The proposed method, which is the bidirectional LSTM (BidLSTM), augments the conventional LSTM to enhance a network model’s performance. The BidLSTM model utilizes two hidden LSTM layers to process data inputs in two directions (i.e., forward and backward) [48,49]. The basic concept of a BidLSTM model is quite simple. It involves duplicating the primary recursive layer of the neural model. In training, the input to the primary layer consists of the actual data, while that of the duplicate layer is a reverse copy of the data. This technique effectively increases the amount of information available to the model. Figure 4 displays the structure of a BidLSTM model. The Keras library in Python provides a wrapper for the bidirectional layers used for developing BidLSTMs. It permits users to decide the merging mode, which determines how the outputs from both directions (i.e., forward and backward) are combined before feeding them to the subsequent layer. The forward hidden layer ($\vec{\;\beta }$), the backward hidden layer ($\overset{\leftarrow }{\;\beta }$), and the output (*o*) of a BidLSTM can be obtained from the following equations [49,50]:

(10) $${\vec{\;\beta }}_{t}=h\left({\hat{W}}_{\alpha \vec{\;\beta }}\alpha_{t}+{\hat{W}}_{\vec{\;\beta }\vec{\;\beta }}{\vec{\;\beta }}_{t-1}+\lambda_{\vec{\;\beta }}\right)$$

(11) $${\overset{\leftarrow }{\;\beta }}_{t}=h\left({\hat{W}}_{\alpha \overset{\leftarrow }{\;\beta }}\alpha_{t}+{\hat{W}}_{\overset{\leftarrow }{\;\beta }\overset{\leftarrow }{\;\beta }}{\overset{\leftarrow }{\;\beta }}_{t+1}+\lambda_{\overset{\leftarrow }{\;\beta }}\right)$$

(12) $$o_{t}={\hat{W}}_{\vec{\;\beta }o}{\vec{\;\beta }}_{t}+{\hat{W}}_{\overset{\leftarrow }{\;\beta }o}{\overset{\leftarrow }{\;\beta }}_{t}+\lambda_{o}$$

where ${\hat{W}}_{\alpha \vec{\;\beta }}$ denotes the forward hidden weight and ${\hat{W}}_{\alpha \overset{\leftarrow }{\;\beta }}$ denotes the backward hidden weight. The terms $\lambda_{\vec{\;\beta }}$ and $\lambda_{\overset{\leftarrow }{\;\beta }}$ signify the forward and backward bias vectors, respectively, while the term h denotes the hidden layer.

It is evident from the literature that bidirectional RNN models perform considerably better than standard models in several research areas, including intrusion detection. In this approach, we evaluated the performance of BidLSTM using the NSL-KDD intrusion detection dataset. The $\chi^{2}$ statistical model was hybridized with the BidLSTM to further improve the model’s performance. We carried out experiments to discover the hyper-parameter values that would result in the best IDS performance metrics. The trained $\chi^{2}$-BidLSTM model consisted of an input layer with 64 neurons, three hidden layers with 32 neurons each, and an output layer with five neurons corresponding to the five class labels. We set the number of epochs to 100, with a range of 0 to 0.05 as the model weights. We defined the loss function in the training process to assess the model weights. Since the study deals with a multi-class classification issue, we chose an algorithmic loss function specified in the Keras library as “categorical_crossentropy”. This loss function measures how the predicted values vary from the actual values. We employed ReLU as the activation function for all layers except the output layer, which used Softmax activation. The model uses an “Adam” optimization algorithm with a learning rate of 0.008. Finally, we fitted the model to the dataset using the “fit” function. We adopted the K-fold cross-validation scheme with the value of K set to 10, to evaluate the model’s performance.

## 4. Experimental Results and Discussion

This section presents the process of implementing $\chi^{2}$-BidLSTM (Algorithm 1) and discusses the experimental results. To investigate the proposed method’s robustness, we evaluated the model’s performance using different metrics such as accuracy, precision, F-score, and false alarm rate (FAR). In addition, this section compares the findings to those of the standard LSTM model and other techniques in the literature. The complexity and runtime analyses of the proposed algorithm are also provided.

### 4.1. Implementation

The proposed method is a feature-selection-based IDS called $\chi^{2}$-BidLSTM. Several tools in the literature can be used to perform this type of experiment. In this study, the Python programming language was utilized to implement the different phases of the proposed method. To be precise, we used Python’s TensorFlow and Keras libraries to implement the various components of the model. All experiments and evaluations were carried out using a personal computer (PC) running on the Windows 10 Operating System (OS), with the following specifications: Intel Core i5-9300H CPU, 8GB Random Access Memory (RAM), NVIDIA GeForce GTX 1050, and a 4GB dedicated GDDR5 VRAM. The implementation was in two phases. The first phase was feature selection with a chi-square statistical model. As mentioned earlier, the NSL-KDD dataset contains 41 training features. However, the dataset contains some irrelevant features that can hinder the performance of a model. To improve the prediction accuracy, we used a $\chi^{2}$ feature selection method to narrow down the features to those most relevant for classification. As shown in Section 1, the $\chi^{2}$ model computes the scores between each feature D[i] and class label *L* and ranks the features in descending order based on their test scores. The result is saved in *SELECTED*. The algorithm finally returns *SELECTED*, containing the list of ranked feature indexes. After ranking all features, a forward best search was performed to select an optimal set, as stated in Section 3.3.1. The search first finds the feature having the highest $\chi^{2}$ test score using the evaluation function v() and appends it to *SELECTED*. The next iteration finds the subsequent feature that achieves the highest score in addition to the features in *SELECTED*. The procedure is repeated until an ideal feature combination is achieved and fed to the classification models to produce the best results.

### 4.2. Results and Discussion

In this section, we present a discussion of the results obtained from all experiments. We performed a total of four separate experiments using the NSL-KDD dataset with 10-fold cross-validation.

#### 4.2.1. Experiment No. 1: Standard LSTM Trained with All 41 Features

In experiment 1, we investigated a standard LSTM model’s performance using all 41 features for 5-class classification (i.e., DoS, Probe, U2R, R2L, and Normal). Table 5 illustrates the confusion matrices used to evaluate the model’s performance, and the results are reported in Table 6 and Table 7.
**Algorithm 1.**$\chi^{2}$-BidLSTM implementation process                      ▹ Obtain the $\chi^{2}$ test scores for each feature using $\chi^{2}$ statistical model                    ▹ Rank(sort) the features in descending order based on their $\chi^{2}$ test scores1:arr← {}2:**for** i ← 1 **to** *n* **do**3:    test_score←chi.sqaured(D[i],L)▹ Compute the $\chi^{2}$ score between features in the dataset *D* and class labels *L*4:    **append** (*i*, test_score) **to**arr5:**end for**6:**rank** the features of arr▹ Sort the features in a descending order based on their $\chi^{2}$ test scores7:**store** the feature scores of arr**to**SELECTED8:**return**SELECTED               ▹ Find the features with the highest test value (v_max) from the ranked features                       ▹ Obtain the best feature subset for training using forward search9:SELECTED← {}10:v_max←−111:SubF← index of D12:**while**SubF != NULL **do**13:    index←NULL14:    **for** i ← 0 **to**SubF length **do**15:         tempfeature_list←(SELECTED∪SubF[i])16:         tempv←v(tempfeature_list)17:         **if** tempv>v_max **then**18:            index ← i19:         **end if**20:    **end for**21:    **if** index == NULL **then**22:        **break**23:    **else**24:        **append** SubF[index] **to** SELECTED25:        **Remove** SubF[index] **from** SubF26:    **end if**27:**end while**28:**return** SELECTED as optimal set               ▹ Model training interface with a K-fold cross-validation using the optimal set29:**for** f = 1 **to** *k* **do**30:    [ ]Training_set = New_List[|V|]31:    [ ]Testing_set = New_List[|V|]                        ▹ Construct the training set32:    **for** m = 1 **to** *k* **do**33:        **if** m == f **then**34:           continue35:        **end if**36:        **for** v = 1 **to** |V| **do**37:           Train[v] + fold[v][m]38:        **end for**39:    **end for**                                  ▹ Construct the testing set40:    **for** v = 1 **to** |V| **do**41:        Test[v] + fold[v][m]42:    **end for**                         ▹ Fit BidLSTM model for training and testing43:    model = BidLSTM()44:    BidLSTM.Fit(Train)                            ▹ Train model with K-1 folds45:    Evaluate model perfomance with remaining Kth folds46:    scores = cross_val_scores()47:    **Return** scores                  ▹ Return the classification accuracy and validation scores48:**end for**49:Test model with an unseen test dataset50:**Return** test scores

As reported in Table 6, the standard LSTM model produces a detection accuracy of 87.26%, a precision of 90.34%, an F-Score of 88.03%, and a false alarm rate of 4.03% for the NSL-KDDTest^+^ dataset. From Table 7, the model produced a 74.49% detection accuracy, 81.53% precision, an F-Score of 75.76%, and a 5.96% false alarm rate for the NSL-KDDTest^−21^ dataset.

#### 4.2.2. Experiment No. 2: BidLSTM Trained with all 41 Features

The second phase of the experiments involved a bidirectional LSTM model trained with all 41 features of the dataset. We evaluated the model’s performance using the confusion matrix and experimental findings shown in Table 8, Table 9 and Table 10.

To obtain a better intuition about the numbers of correctly classified attacks and the misclassification rates, we tabulated the confusion matrix in Table 8 for the two test sets (i.e., NSL-KDDTest^+^ and NSL-KDDTest^−21^). The vertical labels denote the true classes while the horizontal labels represent the predicted classes. As mentioned in Section 3.1, the NSL-KDDTest^+^ dataset contains 22,544 traffic records, out of which 12,833 samples are malicious behaviors (attacks) and 9711 are normal behaviors. Out of the 12,833 attack samples, the BidLSTM model could correctly detect 11,332, producing a detection accuracy of 91.36% from the confusion matrix, a precision of 92.81%, and an F-score of 91.67%. The model misclassified 1501 attack samples, yielding a low false alarm rate of 3.06%. Similarly, the NSL-KDDTest^−21^ test set contains 2152 normal and 9698 attack records. BidLSTM correctly detected 7947 attacks while 1751 were misclassified. Thus, the model achieved 82.05% detection accuracy, 85.91% precision, an F-Score of 82.77%, and a false alarm rate of 4.20% compared to the standard LSTM model. The performances of our proposed approach, BidLSTM, and those of other current techniques using all the 41 features in the NSL-KDD dataset are reported in Table 11.

**Figure 5:**
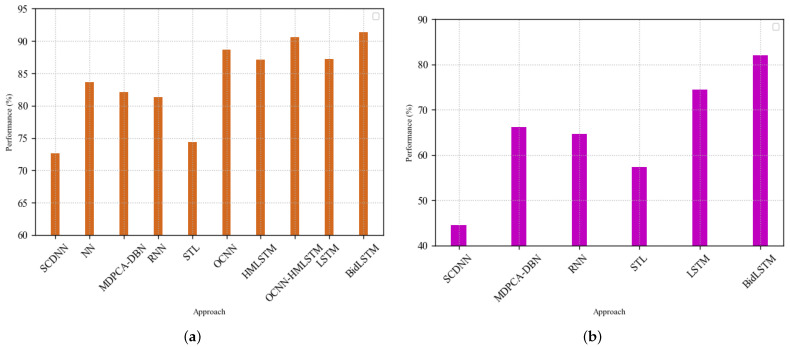
Comparison of results against existing methods on NSL-KDDTest^+^ and NSL-KDDTest^−21^ using all 41 features. (**a**) Performance results on NSL-KDDTest^+^; (**b**) performance results on NSL-KDDTest^−21^.

Based on the results presented in Table 11, our approach shows substantial advantages over the other methods on the NSL-KDD dataset. BidLSTM trained using all 41 features reliably exhibits a greater detection accuracy and a better F-score than the other methods on the two test sets (i.e., NSL-KDDTest^+^ and NSL-KDDTest^−21^), as shown in Figure 5. Additionally, it also has a lower FAR than these approaches, indicating its effectiveness in detecting intrusions.

#### 4.2.3. Experiment No. 3: Standard LSTM Trained with Reduced Features

**Figure 6:**
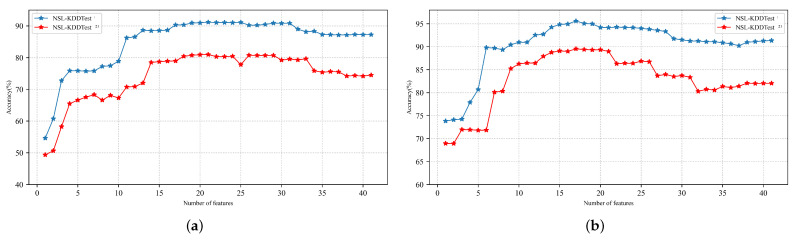
Performance results of different subsets of features on NSL-KDDTest^+^ and NSL-KDDTest^−21^. (**a**) Performance of different subsets on NSL-KDDTest^+^; (**b**) performance of different subsets on NSL-KDDTest^−21^.

In this section, we investigate the performance of the chi-squared feature selection integrated with the standard LSTM model. Using the $\chi^{2}$ statistical model, we achieved different subsets of features. These subsets were fed successively to the standard LSTM classification model for training, and the performance of each subset was recorded as shown in Section 6. The subset of features that produced the best performance results was selected as the optimal set as shown in Figure 6. Table 12 presents the list of features in the chosen subset.

As shown in Table 13, with just 21 features, the model could correctly detect 11,377 malicious records out of the 12,833 records in the NSL-KDDTest^+^ dataset, producing a higher accuracy of 91.16% compared to training the model with all 41 features. Additionally, with the reduced set of features, the LSTM model obtained 91.86% precision, 96.23% specificity, an F-score of 91.32%, and a recall of 91.20%. It also produced a low false alarm rate of 3.77% compared to the standard LSTM trained with all features.

The experimental results from Table 14 indicate that the standard LSTM model trained with the reduced feature set improved the performance by 3.90%.

In the same vein, Table 15 shows that the model improved the performance by 6.49% with the reduced feature set. That is, with the NSL-KDDTest^−21^ dataset, as presented in Table 13, the model accurately detected 7760 attack records out of a total of 9698, achieving a detection accuracy of 80.98% compared to when it was trained with the complete feature set. It obtained precision, specificity, recall and F-score values of 84.95%, 95.49%, 80.97%, and 81.68%, respectively, with a low false alarm rate of 4.51%.

#### 4.2.4. Experiment No. 4: BidLSTM Trained with Reduced Features

In this experiment, we evaluated the performance of the proposed BidLSTM method using a reduced feature set. Similarly to experiment No. 3, we obtained different subsets of features after applying $\chi^{2}$ feature selection. Sequentially, these feature subsets were fed to the BidLSTM model for training and classification. We then selected the subset with the best performance as the optimal feature set, as shown in Table 12.

From Table 16, Table 17 and Table 18, we can observe that the BidLSTM model had higher detection accuracy, precision, specificity, F-score, and recall. It is evident from the results that BidLSTM trained with a reduced set of features improves the performance of BidLSTM trained with all 41 features by 4.26% and 7.50% on the NSL-KDDTest^+^ and NSL-KDDTest^−21^ datasets, respectively. With 17 features, as presented in Table 12, the model could correctly detect 11,976 attack samples out of the 12,833 samples in the NSL-KDDTest^+^ dataset, yielding a greater accuracy of 95.62%. Furthermore, it achieved a higher precision of 95.88%, a specificity of 97.89%, an F-score of 95.65%, and a recall of 95.62%. It produced a 2.11% false alarm rate, which was lower than when the model was trained with a complete feature set. In addition, it can be observed from Table 16 that BidLSTM trained with 17 features could effectively detect 8644 attacks from a total of 9698 attack records in the NSL-KDDTest^−21^ dataset. That is, using the NSL-KDDTest^−21^ test dataset, BidLSTM with the reduced feature set obtained a detection accuracy of 89.55%, with a low false alarm rate of 2.71%, as shown in Table 18. It also achieved 90.75% precision, 97.29% specificity, 89.55% recall, and an F-score of 89.77%. A comparison of the performances of our proposed method and other existing feature reduction methods is shown in Table 19.

**Figure 7:**
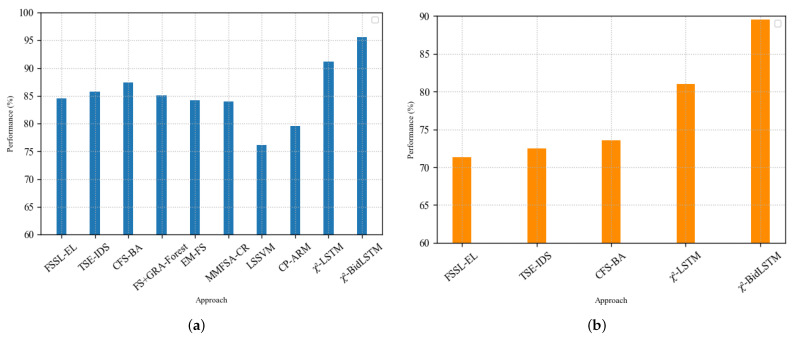
Comparison of results against existing feature selection methods on NSL-KDDTest^+^ and NSL-KDDTest^−21^. (**a**) Performance results on NSL-KDDTest^+^; (**b**) performance results on NSL-KDDTest^−21^.

To broaden the scope of the benchmark, we compared the performance of our $\chi^{2}$-BidLSTM approach to that of earlier studies that used the NSL-KDD Test^+^ and NSL-KDDTest^−21^ datasets. Figure 7 shows the comparison of our results with some of the earlier techniques on these two test sets. The proposed approach, which outperforms other contemporary IDS algorithms, achieved the best detection accuracy based on experimental findings on the NSL-KDD datasets. In addition to having greater detection accuracy, the proposed approach outperformed prior approaches significantly in terms of the false-alarm-rate measure. Our proposed $\chi^{2}$-BidLSTM method achieved greater accuracy of 95.62% and an F-score of 95.65%, with a false alarm rate of 2.11% on the NSL-KDDTest^+^ dataset, using only 17 features. Furthermore, the proposed method obtained an accuracy of 89.55%, an F-score of 89.77%, and a false alarm rate of 2.71%, with just 17 features, according to the experimental findings on the KDDTest^−21^ dataset, which is superior to the $\chi^{2}$-LSTM method and the other existing approaches based on all the performance measures presented in Table 19. The table shows that feature selection improves the performance of both the standard LSTM and BidLSTM models considerably in predicting network intrusion. Chi-square feature selection, compared to other existing feature-selection-based approaches (i.e., PCA, information gain, mutual nformation, CFS, and gain ratio), exhibited superiority in terms of detection accuracy and FAR.

## 5. Model Complexity and Limitations

This subsection addresses the complexity of the proposed $\chi^{2}$-BidLSTM method and the time needed for training and testing. Furthermore, we also present the limitations of the proposed approach.

### 5.1. Time Complexity

We evaluated the time complexity of our proposed $\chi^{2}$-BidLSTM approach with regard to the various units of the model implementation: feature ranking using the $\chi^{2}$ statistical model, optimal feature selection using the forward best search algorithm, and BidLSTM. To obtain the best feature combination set for training, we first used the $\chi^{2}$ statistical model to rank all features in a descending order based on their $\chi^{2}$ test scores, as shown in Section 1. The time complexity is O(n×F), where *n* is the number of classes and *F* is the number of features. After ranking all the features, we used the forward best search algorithm to obtain the optimal feature set for each model. The algorithm begins with an empty set. It searches for the feature with the highest $\chi^{2}$ test score using the evaluation function and appends it to *SELECTED* (see Algorithm Section 1; *lines 9–28*). The algorithm continuously finds the next feature that can achieve the best evaluation score with the feature(s) in *SELECTED* until the desired dimension is reached and no additional features can improve the accuracy. The algorithm’s time complexity is O(D), where *D* is the desired dimension.

The core unit of the approach is the BidLSTM model, which trains two LSTM layers (i.e., the first layer in the forward direction and the second in reverse order). The time complexity for training the forward LSTM layer is $O(\left(QH\right)+\left(QM_{c}B_{s}\right)+\left(HU_{f}\right)+\left(M_{c}B_{s}U_{f}\right))$, where *Q* denotes the total number of output units, *H* represents the total number of hidden layers, and $M_{c}$ represents the total number of memory cell blocks, with $B_{s}$ as the size of the cell blocks $\left(B_{s}>0\right)$. The number of units associated with the memory cells, gates, and hidden units in the forward direction is denoted by $U_{f}$. With an equal time complexity needed to train the reversed order, the time complexity for training the BidLSTM predictive model is $O\left(2\left[\left(QH\right)+\left(QM_{c}B_{s}\right)+\left(HU_{f}\right)+\left(M_{c}B_{s}U_{f}\right)\right]\right)=O\left(W\right)$, where *W* represents the overall weights necessary for the network model. Hence, the total computational complexity of the proposed $\chi^{2}$-BidLSTM with respect to time is O((n×F)+D+W).

### 5.2. Execution Time Analysis

**Figure 8:**
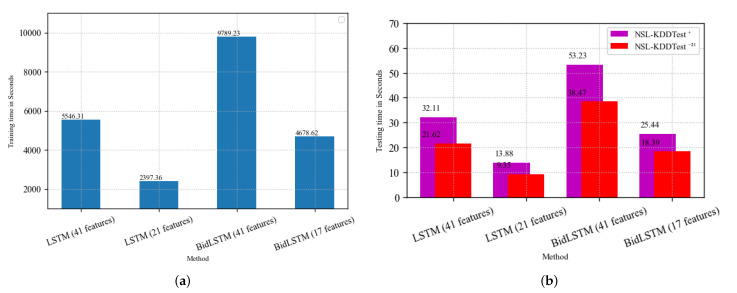
Training and testing times of the methods used in the study. (**a**) Training times of the various methods in seconds; (**b**) testing times of the various methods in seconds.

In this subsection, we analyze the testing and training times of the models used in this study. To ensure a fair and accurate analysis, we performed all the experiments using an Intel Core i5 PC with an 8 GB memory. The training and testing times of the various methods are reported in Figure 8a and Figure 8b, respectively. From Figure 8a, it can be seen that the BidLSTM approach with the complete set of features requires more time (9789.24 s) to train than the standard LSTM model (5546.31 s). The reason is that BidLSTM trains two LSTMs with an entry shape of a dimensional matrix of the data length and the number of features used. In this domain, the most important characteristic of a model is its ability to accurately, actively, and effectively detect network intrusion. As such, there is a trade-off between training time and performance. Therefore, the standard LSTM model may require a shorter training time, but the BidLSTM model exhibits better accuracies in detecting intrusions. From Figure 8, it is evident that feature selection reduces the training and testing times of both methods considerably. With the reduced set of features, the standard LSTM model requires a training time of 2397.36 s, whiles it takes 4678.62 s to train the BidLSTM model. Thus, feature selection not only improves the performances of the various models but also minimizes the computational times of the models.

### 5.3. Limitations

The experimental findings demonstrate that the proposed method can efficiently detect network intrusions. However, more study in this domain is still required to improve the overall performance of the proposed approach further. The proposed $\chi^{2}$-BidLSTM model has a higher complexity and needs more training time than the standard LSTM model, as demonstrated by the complexity and runtime analyses of the model. In the real-world network scenario of computer systems, new forms of intrusions emerge continually, which may not be captured by the NSL-KDD dataset. The absence of emerging novel attacks in the dataset can make it difficult for the proposed method to adapt to recently emerging attacks in a network system. These are the primary limitations of the $\chi^{2}$-BidLSTM-based intrusion detection model.

## 6. Conclusions and Future Directions

### 6.1. Conclusions

Even though various machine learning methods have been proposed to enhance the performance of IDSs, most existing intrusion detection methods still struggle to achieve good performance. This study offers a new IDS approach called $\chi^{2}$-BidLSTM that integrates the chi-square ($\chi^{2}$) statistical model with a bidirectional long short-term memory (BidLSTM) model. We used the $\chi^{2}$ statistical model to reduce the dataset to the optimal set, to handle the imbalance and high dimensionality of the data. The NSL-KDD dataset with 10-fold cross-validation was used to evaluate the performance of our proposed $\chi^{2}$-BidLSTM approach, and the results were compared to other existing intrusion detection approaches. The experimental findings indicated that the proposed $\chi^{2}$-BidLSTM method improved the intrusion detection accuracies of the standard LSTM and BidLSTM models by 3.90% and 4.26%, respectively, on the NSL-KDDTest^+^ test set, and by 6.49% and 7.50%, respectively, on the NSL-KDDTest^−21^ test set. Compared with previously existing techniques that utilize feature selection, the proposed approach achieved higher detection accuracy and F-scores on the two test datasets, while maintaining lower false alarm rates. Furthermore, the proposed $\chi^{2}$-BidLSTM method exhibited good performance in detecting minority attack types such as User-to-Root (U2R) and Remote-2-Local (R2L) attacks compared to the other techniques, indicating the robustness and effectiveness of our proposed approach.

### 6.2. Future Directions

The future direction of this study is to explore more feature selection algorithms to further improve the intrusion detection rate of our model. Additionally, as mentioned in Section 5.3, although the proposed approach has a higher intrusion detection accuracy with a low false alarm rate (FAR), the approach has a significant execution-time cost due to the BidLSTM deep network architecture and computations within the LSTM memory cells. Hence, we intend to investigate how to reduce the computational complexity of the proposed method further while maintaining high detection accuracy and a low false alarm rate. We also plan to investigate the performance of our approach with the latest intrusion detection datasets with real-world traffic, such as the UNSW-NB15 and CIC-IDS2017 datasets.

## Figures and Tables

**Table 1:** Traffic sample breakdown of the NSL-KDD dataset.

	Class	Number of Samples		
**Attack Type**		KDDTrain^+^	KDDTest^+^	KDDTest^−21^
	DoS	45,927	7458	4342
	Probe	11,656	2421	2402
	U2R	52	200	200
	R2L	995	2754	2754
	Normal	67,343	9711	2152
**Total**		125,973	22,544	11,850

**Table 2:** List of all 41 features in the NLS-KDD dataset.

No.	Feature	Code	No.	Feature	Code
01	duration	$F_{01}$	22	is_guest_login	$F_{22}$
02	protocol_type	$F_{02}$	23	count	$F_{23}$
03	service	$F_{03}$	24	srv_count	$F_{24}$
04	flag	$F_{04}$	25	serror_rate	$F_{25}$
05	src_bytes	$F_{05}$	26	srv_error_rate	$F_{26}$
06	dst_bytes	$F_{06}$	27	rerror_rate	$F_{27}$
07	land	$F_{07}$	28	srv_rerror_rate	$F_{28}$
08	wrong_fragment	$F_{08}$	29	same_srv_rate	$F_{29}$
09	urgent	$F_{09}$	30	diff_srv_rate	$F_{30}$
10	hot	$F_{10}$	31	srv_diff_host_rate	$F_{31}$
11	num_failed_logins	$F_{11}$	32	dst_host_count	$F_{32}$
12	logged_in	$F_{12}$	33	dst_host_srv_count	$F_{33}$
13	num_compromised	$F_{13}$	34	dst_host_same_srv_rate	$F_{34}$
14	root_shell	$F_{14}$	35	dst_host_diff_srv_rate	$F_{35}$
15	su_attempted	$F_{15}$	36	dst_host_same_src_port_rate	$F_{36}$
16	num_root	$F_{16}$	37	dst_host_srv_diff_host_rate	$F_{37}$
17	num_file_creations	$F_{17}$	38	dst_host_serror_rate	$F_{38}$
18	num_shells	$F_{18}$	39	dst_host_srv_serror_rate	$F_{39}$
19	num_access_files	$F_{19}$	40	dst_host_rerror_rate	$F_{40}$
20	num_outbound_cmds	$F_{20}$	41	dst_host_srv_rerror_rate	$F_{41}$
21	is_host_login	$F_{21}$			

**Table 3:** Categories of the various attack types.

Class	Types of Attacks	
	**Training Set**	**Testing Set**
DoS	smurf, neptune, land, back, teardrop, pod	land, pod, apache2, processtable, neptune, smurf, worm, udpstorm, back, mailbomb, teardrop
Probe	satan, nmap, portsweep, ipsweep	portsweep, satan, nmap, ipsweep, saint, mscan
U2R	perl, loadmodule, buffer-overflow, rootkit	ps, rootkit, sqlattack, buffer-overflow, xterm, loadmodule, perl
R2L	imap, warezmaster, fpt-write, warezclient, spy, phf, multihop, guess-passwd	warezmaster, snmpguess, phf, xsnoop, httptunnel, snmpgetattack, sendmail, warezclient, fpt-write, named, xlock, spy, imap, guess-passwd, multihop
Normal	normal	normal

**Table 4:** Computation of chi-square test scores.

	Normal Class	Attack Class	Total
$F_{i}$ occur	*n*	*a*	*n* + *a* = φ
$F_{i}$ do not occur	ν	ν	ν+ρ=m−φ
Total	n+ν=δ	a+ρ=m−δ	*m*

**Table 5:** Confusion matrix for standard LSTM model trained with all 41 features.

		Predicted Label					Predicted Label				
		Normal	DoS	Probe	R2L	U2R	Normal	DoS	Probe	R2L	U2R
**True Label**	Normal	8958	4	729	9	11	1685	11	433	8	15
	DoS	408	6441	538	6	65	652	3064	527	6	93
	Probe	241	26	2114	18	22	258	23	2062	25	34
	R2L	184	5	498	2067	0	439	7	379	1929	0
	U2R	45	2	61	0	92	51	3	58	1	87
**Test Set**	**NSL-KDDTest^+^**						**NSL-KDDTest^−21^**				

**Table 6:** Standard LSTM performance on NSL-KDDTest^+^ using all 41 features.

Class Label	Performance Results (%)				
	Precision	Specificity	Recall	FAR	F-Score
DoS	99.43	99.75	86.36	0.25	92.44
Probe	53.65	90.93	87.32	9.07	66.47
R2L	98.43	99.83	75.05	0.17	85.17
U2R	48.42	99.56	46.00	0.44	47.18
Normal	91.07	93.16	92.25	6.84	91.66

**Table 7:** Standard LSTM performance on NSL-KDDTest^−21^ using all 41 features.

Class Label	Performance Results (%)				
	Precision	Specificity	Recall	FAR	F-Score
DoS	98.58	99.41	70.57	0.59	82.26
Probe	59.61	85.21	85.85	14.79	70.36
R2L	97.97	99.56	70.04	0.44	81.69
U2R	37.99	98.78	43.50	1.22	40.56
Normal	54.62	85.56	78.30	14.44	64.35

**Table 8:** Confusion matrix of BidLSTM model trained with all 41 features.

		Predicted Label					Predicted Label				
		Normal	DoS	Probe	R2L	U2R	Normal	DoS	Probe	R2L	U2R
**True Label**	Normal	9264	1	435	3	8	1776	2	364	2	8
	DoS	321	6738	343	3	53	426	3657	221	2	36
	Probe	191	9	2216	0	5	195	17	2165	10	15
	R2L	173	0	311	2270	0	446	0	281	2027	0
	U2R	39	0	53	0	108	35	0	60	7	98
**Test Set**	**NSL-KDDTest^+^**						**NSL-KDDTest^−21^**				

**Table 9:** BidLSTM performance on NSL-KDDTest^+^ using all 41 features.

Class Label	Performance Results (%)				
	Precision	Specificity	Recall	FAR	F-Score
DoS	99.85	99.93	90.34	0.07	94.86
Probe	65.99	94.32	91.53	5.68	76.69
R2L	99.74	99.97	82.43	0.03	90.26
U2R	62.07	99.70	54.00	0.30	57.75
Normal	92.75	94.36	95.40	5.64	94.06

**Table 10:** BidLSTM performance on NSL-KDDTest^−21^ using all 41 features.

Class Label	Performance Results (%)				
	Precision	Specificity	Recall	FAR	F-Score
DoS	99.48	99.75	84.22	0.25	91.22
Probe	70.04	90.20	90.13	9.80	78.83
R2L	98.97	99.77	73.60	0.23	84.42
U2R	62.42	99.49	49.00	0.51	54.90
Normal	61.71	88.64	82.53	11.36	70.62

**Table 11:** Performance comparison against existing methods in the literature using all 41 features (N/A denotes not available).

Approach	Performance (%)					
	NSL-KDDTest^+^			NSL-KDDTest^−21^		
	Accuracy	F-Score	FAR	Accuracy	F-Score	FAR
SCDNN [51]	72.64	N/A	27.36	44.55	N/A	55.45
NN [52]	83.67	83.28	23.47	N/A	N/A	N/A
MDPCA-DBN [53]	82.08	81.75	2.62	66.18	74.87	13.06
RNN [54]	81.29	79.25	12.42	64.67	N/A	N/A
STL [55]	74.38	N/A	7.21	57.34	N/A	15.06
OCNN [56]	88.67	89.78	11.89	N/A	N/A	N/A
HMLSTM [56]	87.11	88.40	12.20	N/A	N/A	N/A
OCNN-HMLSTM [56]	90.61	91.46	8.86	N/A	N/A	N/A
Standard LSTM	87.26	88.03	4.03	74.49	75.76	5.96
BidLSTM	91.36	91.67	3.06	82.05	82.77	4.20

**Table 12:** The selected optimal set of features.

Method	Feature Code	Number of Features
Standard LSTM	[$F_{02}$, $F_{03}$, $F_{04}$, $F_{05}$, $F_{06}$, $F_{08}$, $F_{10}$, $F_{13}$, $F_{14}$, $F_{22}$, $F_{24}$, $F_{25}$, $F_{27}$, $F_{28}$, $F_{29}$, $F_{31}$, $F_{33}$, $F_{34}$, $F_{38}$, $F_{40}$, $F_{41}$]	21
BidLSTM	[$F_{02}$, $F_{03}$, $F_{04}$, $F_{05}$, $F_{06}$, $F_{08}$, $F_{10}$, $F_{13}$, $F_{14}$, $F_{22}$, $F_{24}$, $F_{25}$, $F_{27}$, $F_{28}$, $F_{29}$, $F_{31}$, $F_{33}$]	17

**Table 13:** Confusion matrix of standard LSTM model trained with 21 features.

		Predicted Label					Predicted Label				
		Normal	DoS	Probe	R2L	U2R	Normal	DoS	Probe	R2L	U2R
**True Label**	Normal	9175	7	505	16	8	1836	12	269	21	14
	DoS	375	6806	215	9	53	405	3343	493	13	88
	Probe	111	156	2120	21	13	178	33	2143	48	0
	R2L	322	0	100	2325	7	328	9	266	2128	23
	U2R	68	0	0	6	126	35	0	19	0	146
**Test Set**	**NSL-KDDTest^+^**						**NSL-KDDTest^−21^**				

**Table 14:** Standard LSTM performance results on NSL-KDDTest^+^ using 21 features.

Class Label	Performance Results (%)				
	Precision	Specificity	Recall	FAR	F-Score
DoS	97.66	98.92	91.26	1.08	94.35
Probe	72.11	95.93	87.57	4.07	79.09
R2L	97.81	99.74	84.42	0.26	90.63
U2R	60.87	99.64	63.00	0.36	61.92
Normal	91.28	93.17	94.48	6.83	92.85

**Table 15:** Standard LSTM performance results on NSL-KDDTest^−21^ using 21 features.

Class Label	Performance Results (%)				
	Precision	Specificity	Recall	FAR	F-Score
DoS	98.41	99.28	76.99	0.72	86.39
Probe	67.18	88.92	89.22	11.08	76.65
R2L	96.29	99.10	77.27	0.90	85.74
U2R	53.87	98.93	73.00	1.07	62.00
Normal	66.00	90.25	85.32	9.75	74.42

**Table 16:** Confusion matrix of BidLSTM model trained with 17 features.

		Predicted Label					Predicted Label				
		Normal	DoS	Probe	R2L	U2R	Normal	DoS	Probe	R2L	U2R
**True Label**	Normal	9580	0	116	5	10	1968	25	150	0	9
	DoS	261	7018	152	0	27	286	3912	94	7	43
	Probe	127	1	2293	0	0	52	84	2258	8	0
	R2L	142	5	106	2501	0	276	0	175	2303	0
	U2R	34	2	0	0	164	13	7	0	9	171
**Test Set**	**NSL-KDDTest^+^**						**NSL-KDDTest^−21^**				

**Table 17:** BidLSTM performance results on NSL-KDDTest^+^ using 17 features.

Class Label	Performance Results (%)				
	Precision	Specificity	Recall	FAR	F-Score
DoS	99.89	99.95	94.10	0.05	96.91
Probe	85.98	98.14	94.71	1.86	90.13
R2L	99.80	99.97	90.81	0.03	95.10
U2R	81.59	99.83	82.00	0.17	81.80
Normal	94.44	95.61	98.65	4.39	96.50

**Table 18:** BidLSTM performance results on NSL-KDDTest^−21^ using 17 features.

Class Label	Performance Results (%)				
	Precision	Specificity	Recall	FAR	F-Score
DoS	97.12	98.45	90.10	1.55	93.48
Probe	84.35	95.57	94.00	4.43	88.92
R2L	98.97	99.74	83.62	0.26	90.65
U2R	76.68	99.55	85.50	0.44	80.85
Normal	75.84	93.53	91.45	6.47	82.92

**Table 19:** Comparison of results against existing feature-selection-based algorithms on NSL-KDDTest^+^ and NSL-KDDTest^−21^.

Approach	Feature Selection Method	Number of Features	Performance (%)					
			**NSL-KDDTest^+^**			**NSL-KDDTest^−21^**		
			**Accuracy**	**F-score**	**FAR**	**Accuracy**	**F-score**	**FAR**
FSSL-EL [57]	PCA	20	84.54	N/A	5.31	71.29	N/A	20.35
TSE-IDS [58]	Hybrid	37	85.80	N/A	11.70	72.52	N/A	18.00
CFS-BA [29]	CFS	10	87.37	N/A	3.19	73.57	N/A	12.92
FS+GRA-Forest [59]	Information Gain	32	85.06	85.10	12.20	N/A	N/A	N/A
EM-FS [30]	Gain Ratio	35	84.25	N/A	2.79	N/A	N/A	N/A
MMFSA-CR [60]	Hybrid	19	83.98	N/A	N/A	N/A	N/A	N/A
LSSVM [61]	Mutual Information	18	76.20	76.10	3.90	N/A	N/A	N/A
CP-ARM [62]	Hybrid	11	79.60	79.50	3.50	N/A	N/A	N/A
$\chi^{2}$-LSTM	Chi-Square	21	91.16	91.32	3.77	80.98	81.68	4.51
$\chi^{2}$-BidLSTM	Chi-Square	17	95.62	95.65	2.11	89.55	89.77	2.71

## Data Availability

The dataset used or analyzed during the current study is publicly available for use from the University of New Brunswick (UNB) data repository. It can be accessed using the following url: Available online: https://www.unb.ca/cic/datasets/nsl.html (accessed on 10 January 2022).

## Abbreviations

The following abbreviations are used in this study:

IDS	Intrusion Detection System
NIDS	Network Intrusion Detection System
LSTM	Long Short-Term Memory
BidLSTM	Bidirectional Long Short-Term Memory
NSL-KDD	New Security Laboratory Knowledge Discovery Data
DL	Deep Learning
ML	Machine Learning
DNN	Deep Neural Network
RNN	Recurrent Neural Networks
ICT	Information and Communication Technology
PCA	Principal Component Analysis
EL	Ensemble Learning
FSSL-EL	Fuzzy-Based Semi-Supervised Learning with Ensemble Learning
TSE-IDS	Two-Stage Ensemble Intrusion Detection System
CFS-BA	Correlation-Based Feature Selection with Bat Algorithm
FS	Feature Selection
GRA	Greedy Randomized Adaptive
EM-FS	Ensemble Model with Feature Selection
MMFSA	Multi-Measure Feature Selection Algorithm
LSSVM	Least Square Support Vector Machine
CP-ARM	Central Points with Association Rule Mining
